# A Technological Review of Digital Twins and Artificial Intelligence for Personalized and Predictive Healthcare

**DOI:** 10.3390/healthcare13141763

**Published:** 2025-07-21

**Authors:** Silvia L. Chaparro-Cárdenas, Julian-Andres Ramirez-Bautista, Juan Terven, Diana-Margarita Córdova-Esparza, Julio-Alejandro Romero-Gonzalez, Alfonso Ramírez-Pedraza, Edgar A. Chavez-Urbiola

**Affiliations:** 1 Departamento de Investigación, Universidad de Investigación y Desarrollo-UDI, Cra. 9 No. 10-40, San Gil 684031, Santander, Colombia; 2 Departamento de Investigación, Fundación Universitaria de San Gil—Unisangil, Km2 vía San Gil—Charalá, San Gil 684031, Santander, Colombia; jramirez@unisangil.edu.co; 3 CICATA-Qro, Instituto Politecnico Nacional, Querétaro 76090, Mexico; jrtervens@ipn.mx (J.T.); pedro.ramirez@secihti.mx (A.R.-P.); eachavezu@ipn.mx (E.A.C.-U.); 4 Facultad de Informática, Universidad Autónoma de Querétaro, Querétaro 76230, Mexico; diana.cordova@uaq.mx (D.-M.C.-E.); julio.romero@uaq.mx (J.-A.R.-G.); 5 Secretaría de Ciencia, Humanidades, Tecnología e Innovación SECIHTI, IxM, Alvaro Obregón, Mexico City 03940, Mexico

**Keywords:** big data analysis, digital health, digital twins, health innovation, real-time communication

## Abstract

Digital transformation is reshaping the healthcare field by streamlining diagnostic workflows and improving disease management. Within this transformation, Digital Twins (DTs), which are virtual representations of physical systems continuously updated by real-world data, stand out for their ability to capture the complexity of human physiology and behavior. When coupled with Artificial Intelligence (AI), DTs enable data-driven experimentation, precise diagnostic support, and predictive modeling without posing direct risks to patients. However, their integration into healthcare requires careful consideration of ethical, regulatory, and safety constraints in light of the sensitivity and nonlinear nature of human data. In this review, we examine recent progress in DTs over the past seven years and explore broader trends in AI-augmented DTs, focusing particularly on movement rehabilitation. Our goal is to provide a comprehensive understanding of how DTs bolstered by AI can transform healthcare delivery, medical research, and personalized care. We discuss implementation challenges such as data privacy, clinical validation, and scalability along with opportunities for more efficient, safe, and patient-centered healthcare systems. By addressing these issues, this review highlights key insights and directions for future research to guide the proactive and ethical adoption of DTs in healthcare.

## 1. Introduction

In recent years, digitization has become a priority in the healthcare sector, largely due to its potential to streamline diagnostic processes and improve disease management [1]. A critical area within healthcare involves providing ongoing medical assistance and effective rehabilitation for a wide range of conditions, including chronic diseases, post-operative recovery, and neurological impairments. These challenges often require highly personalized approaches and continuous monitoring scenarios where early and precise intervention is crucial for improving patient outcomes [2,3]. Consequently, the integration of modern health technologies has become essential for enhancing diagnostic accuracy and accelerating treatment decisions.

One promising solution in this regard is the use of Digital Twins (DTs). A DT serves as a virtual representation of a physical object, system, or even a person that is continually updated with real-time data from sensors. In healthcare, DTs integrate clinical, demographic, and biometric data to create detailed patient profiles, effectively acting as virtual avatars. By simulating and analyzing patient-specific scenarios, DTs empower healthcare professionals to predict treatment outcomes and personalize interventions. They are increasingly seen as a new frontier for personalized medicine [4].

**Figure 1:**
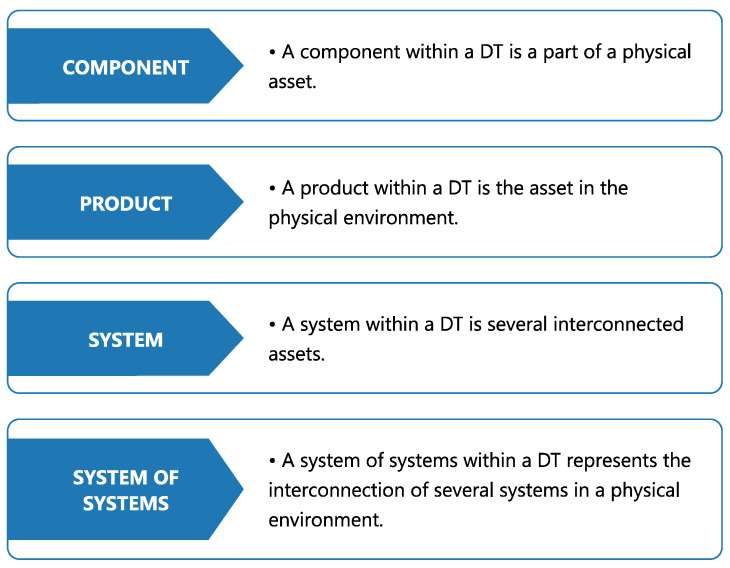
Hierarchical structure of a DT in a system, according to [5].

Before delving into healthcare applications specifically, it is useful to consider how DTs are represented within a broader hierarchical framework [5]. As illustrated in Figure 1, four layers of physical representation are typically defined. The foundation begins with a basic component, either simple or complex, that is later combined with others to form a more advanced product. Multiple products can then be integrated to build an entire system capable of interacting with other systems in real-world settings.

In addition to healthcare, DTs are widely used in the industrial and commercial sectors to reduce costs, mitigate risks, and optimize performance by performing simulations in virtual environments. Regardless of whether the technology is in the design, development, or testing phase, DTs operate within a Digital Twin Environment (DTE). This review distinguishes three main types of DTs based on their stage of implementation:

- Digital Twin Prototype (DTP): Developed before a physical product exists. It enables rapid prototyping and testing of design concepts, materials, and predicted behaviors in a virtual setting [6].
- Digital Twin Instance (DTI): Created for an already existing physical product. A DTI establishes a real-time bidirectional communication link between the physical and virtual domains, allowing for continuous monitoring, validation, and updates [7].
- Digital Twin Aggregation (DTA): DTAs focus on analyzing large-scale data from physical products by leveraging intelligent capabilities to optimize design, monitor performance, and draw data-driven conclusions about product functionality and usage [8].

Since 2021, DTs have gained momentum in operational and production environments. When integrated with the Internet of Things (IoT), Machine Learning (ML), and Artificial Intelligence (AI), DTs facilitate real-time data synchronization and advanced analyses such as pattern recognition and predictive modeling [9]. By using AI-based methods for data acquisition, processing, and ML, these systems can approximate human reasoning and decision-making to a remarkable degree [10].

Historically, two related technologies, *digital models* and *digital shadows*, set the stage for DTs; digital models do not communicate with the physical world, requiring manual updates, while digital shadows maintain only unidirectional data flow from the physical world to the virtual realm [8]. In contrast, DTs enable ongoing *bidirectional* communication, supporting iterative improvements and real-time adjustments.

**Figure 2:**
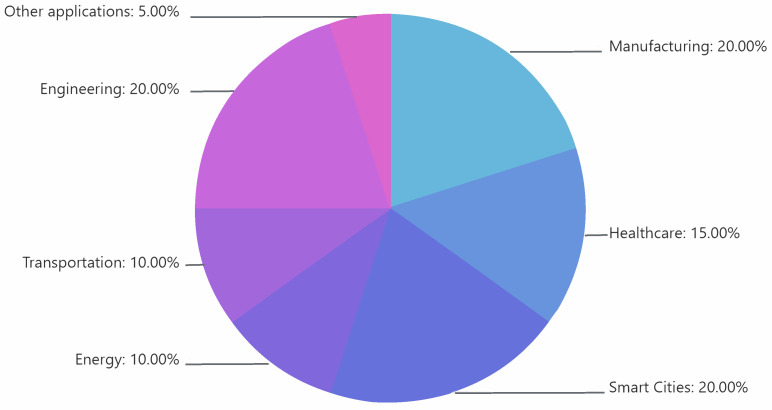
Distribution of publication trends on digital twins and artificial intelligence across various sectors. The pie chart represents the percentage of publications in seven fields.

Figure 2 illustrates the cross-sector adoption of DTs and AI, showing a growing trend across various fields.

In manufacturing, DTs and AI aid in predicting failures and optimizing production, thereby reducing downtime and operational costs [10]. In healthcare, these technologies can support disease diagnosis and treatment planning and assist in designing more efficient healthcare facilities [11]. Meanwhile, the push toward smart cities has led researchers to create city-wide DTs to study traffic flow, public safety, and sustainability [12].

Despite their broad applicability, the level of DT integration varies widely across industries. Many companies remain in the early stages of DT adoption, typically using 3D simulations with some real-world data overlays. Greater challenges arise when trying to synchronize real-time data, which requires robust sensor networks, secure data handling, and reliable connectivity [8].

Although application steps are often straightforward in industrial contexts [13], healthcare presents additional complexity. In medicine, data collection and patient experimentation must meet stringent ethical, safety, and regulatory standards. Moreover, human biology introduces layers of variability and nonlinearity that complicate data interpretation. Systems must account for numerous clinical variables in order to accurately emulate human behaviors in virtual spaces. While current medical devices that capture physiological data can feed these systems, substantial analytical work is required to translate raw data into reliable diagnoses, predictions, and therapeutic plans.

Each DT project generally follows a progression from concept to realization, as outlined in [14]:

1. Mental representation: Formulating an idea of the envisioned product or system.
2. Virtual representation: Creating a computer-generated model to replicate behavior under real-world conditions.
3. Physical realization: Deploying the optimized design into the real environment.
4. Parameter definition: Specifying which characteristics are transferable between physical and virtual entities.
5. Connection features: Enabling synchronization between the physical and virtual worlds.
6. Data analysis methods: Applying techniques in the virtual environment to interpret and leverage collected data.
7. Integrated simulation: Running virtual experiments that inform physical processes and vice versa.
8. Process improvement: Refining design, enhancing performance, and building large-scale datasets over time.
9. Ethical and legal considerations: Ensuring data privacy, security, and regulatory compliance.

In healthcare, adopting DTs poses both significant challenges and transformative opportunities. Human variability, ethical constraints, and the need for rigorous clinical validation all demand careful methodological approaches; yet, DTs move beyond traditional diagnostics, allowing researchers and clinicians to run sophisticated simulations in a risk-free virtual space. This is particularly valuable in areas such as movement rehabilitation, where they can help to identify optimal treatment strategies by simulating patient-specific scenarios, offering an unprecedented ability to tailor interventions and predict outcomes with greater precision. The capacity of DTs to create high-fidelity dynamic representations of individual patients fundamentally reshapes the landscape of personalized medicine. By integrating multimodal data from wearables, medical imaging, and electronic health records, DTs construct comprehensive virtual models that mirror a patient’s physiological state and disease progression in real time. This enables not only personalized treatment plans but also sophisticated predictive medicine, allowing for early intervention and proactive management of health conditions before critical deterioration. Furthermore, DTs facilitate a deeper understanding of complex biological systems by simulating intricate interactions between organs and body systems, unraveling mechanisms of disease and response to therapy in ways previously unattainable. In the critical field of rehabilitation, this translates into the ability to virtually test the impact of different exercises, assistive devices, or surgical approaches on the unique biomechanics and recovery trajectory of a patient, helping to optimizing functional restoration and minimizing adverse events. This groundbreaking capability holds the profound promise of revolutionizing patient care, ushering in an era where treatment is not just reactive but precisely predictive, personalized, and profoundly impactful.

This study aims to examine the core aspects of DT technology as it applies to healthcare, exploring both the foundational methods and the advanced techniques currently shaping the field. By identifying the challenges and opportunities presented by DTs in healthcare, we can work toward an efficient, safe, and patient-centered care model that leverages the full potential of these emerging technologies.

## 2. Methods

The methodology for this review consists of two main phases, each designed to capture both recent advances and longer-term trends in the integration of Digital Twins (DTs) and Artificial Intelligence (AI) within healthcare.

### 2.1. Phase 1: Recent Technological Progress (Past Seven Years)

In the initial phase of this study, a targeted examination of publications from the last seven years (2018–2024) was carried out. This observation window was strategically selected to account for the rapid advances in technology. Although an initial 5-year period was considered sufficient due to the significant progress typically observed within such a time frame, the scope was expanded to include two additional years (2018–2019). This extension allowed for a more comprehensive understanding of the preceding trends and the developmental trajectory that led to the most recent advances. The primary goal was to highlight notable technological developments integrating Digital Twins (DTs) with Artificial Intelligence (AI). The focus was on how these advances have been applied in various medical procedures, including screening, diagnosis, monitoring, and rehabilitation, and the degree to which they can influence or improve clinical outcomes.

#### 2.1.1. Search Strategy

We performed systematic searches in major academic databases including Google Scholar, PubMed, Scopus, and IEEE Xplore using combinations of keywords:

- (“Rehabilitation Robotics” AND “Technology”)
- (“Digital Twin” OR “Digital Twins”)
- (“Digital Twins in Healthcare” OR “Digital Twins” AND “Healthcare”).

**Figure 3:**
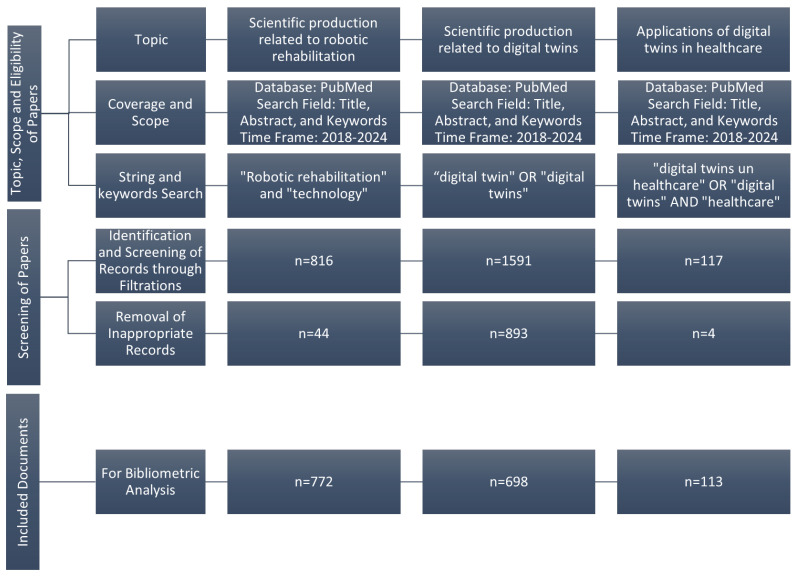
PRISMA flow diagram.

We restricted our search to English-language publications, reflecting its status as the predominant language for scientific communication. Studies that did not directly relate to healthcare or medical applications were excluded. Figure 3 shows the PRISMA flow diagram used to identify appropriate manuscripts.

#### 2.1.2. Selection and Screening

Initial screening involved title and abstract reviews to identify the relevance of each publication to AI-based or DT-based approaches. We then performed a full-text assessment of the remaining articles to ensure that they met the following inclusion criteria:

- The study explicitly integrates AI with DTs in a healthcare or clinical context.
- The research focuses on improving disease screening, diagnosis, or patient monitoring and rehabilitation.
- The publication clearly reports technical features or performance metrics (e.g., accuracy, sensitivity, specificity) of the implemented system.

Studies were excluded if they primarily addressed AI methods not related to healthcare or if full-text versions were unavailable in English. Articles focusing on theoretical or conceptual frameworks without application-level detail were also set aside in order to maintain the review’s practical orientation.

### 2.2. Phase 2: Bibliometric Analysis (2018–2024)

In the second phase, we employed PubMed in conjunction with the Biblioshiny tool of the Bibliometrix R package 4.0.0 [15] to perform a structured bibliometric analysis. The search strategy again prioritized English-language papers related to DTs and AI within healthcare. After metadata extraction, the Bibliometrix package was used to quantify annual publication patterns, identify widely cited works and influential authors, and visualize keyword co-occurrence to detect major research themes and emergent subfields. Insights from Phase 2 were then juxtaposed with the findings from Phase 1 to allow for a unified understanding of how DTs and AI have evolved in medical research and practice.

### 2.3. Research Questions

Drawing on both phases, this review is guided by three central research questions (RQs):

- RQ1: What are the leading technologies used as medical support in diagnostic or monitoring processes involving DTs and AI?
- RQ2: What are the main characteristics of DT applications in the healthcare sector, and how have these been integrated with AI methods?
- RQ3: What benefits can DT and AI offer for future healthcare applications, particularly regarding personalized treatment and predictive analytics?

Through these questions, we aim to systematically assess the current capabilities of AI-driven DTs in healthcare, identify major research gaps, and propose future directions for the field.

## 3. Progress of the Last Seven Years

In recent years, AI-supported Digital Twins (DTs) have emerged as a compelling avenue for advancing healthcare solutions. Their potential spans everything from early diagnosis to rehabilitation, enabling patient-specific treatment plans in virtual spaces before real-world application [16]. By creating tailored virtual environments, DTs can facilitate the simulation of complex clinical cases without placing patients at unnecessary risk while also providing robust datasets to improve AI algorithms. Notably, these AI-driven tools can expedite the development of cutting-edge devices and clinical workflows by reducing reliance on physical prototypes and manual testing.

A noteworthy trend involves the integration of soft robotics into DTs, initially manifesting as Digital Twin Instances (DTIs) for implantable or wearable assistive devices [17]. As these technologies become more sophisticated over time, they are expected to transition into *Digital Twin Aggregation (DTA)* roles, especially in surgical and high-stakes therapeutic contexts. This transition will likely deepen the impact of DTs in mainstream clinical care, where real-time data and feedback loops are critical for personalized treatment and rapid adaptation.

Table 1 summarizes a selection of recent studies that illustrate this progression in digital twin research. These works span applications such as robotic exoskeletons for limb rehabilitation, AI-driven diagnostic support for cancer detection, and intelligent systems for emergency symptom triage. Although the specifics differ, ranging from software tools such as Matlab/Simulink and SolidWorks to algorithms for control strategy such as DDPG-PSO, each study underscores how DTs can enhance real-time monitoring, improve predictive modeling, and enable adaptive interventions in healthcare settings.

Collectively, these implementations have produced promising outcomes. For instance, several prototypes have shown the ability to reduce design mass by nearly half while retaining functionality, while others have demonstrated sub-0.05 tracking error for limb movements in exoskeletons. Early-stage results also suggest improved diagnostic accuracy exceeding 80% in preliminary trials [21] and more streamlined therapeutic processes through immersive virtual environments [25]. Such advancements highlight the value of DTs in lowering development costs, shortening the time to clinical validation, and improving patient engagement and rehabilitation adherence.

Despite these successes, there is a consensus that DT technologies in healthcare are not yet fully mature or clinically validated for large-scale deployment. Many of the highlighted systems still require further development:

- Larger and more diverse datasets: Robust and high-quality data are essential for improving AI model generalizability and confidence in patient-specific predictions.
- More extensive testing and clinical trials: Regulatory bodies and healthcare providers demand evidence from controlled studies demonstrating consistent and reliable performance.
- Greater integration of real-world and virtual systems: Truly seamless bidirectional communication remains a challenge, particularly when attempting real-time synchronization between patient data and virtual models.

Addressing these challenges will be critical to transitioning DT systems from research prototypes to standard clinical practice. Future initiatives may benefit from multi-center collaborations, improved sensor technologies, and standardized protocols that ensure data integrity and patient safety. As the field evolves, the integration of AI and DTs has the potential to revolutionize healthcare delivery, making personalized medicine, predictive diagnostics, and adaptive rehabilitation increasingly feasible.

## 4. Bibliometric Analysis from 2018 to 2024

To understand the research field and recent developments, we performed a bibliometric analysis covering the period from 2018 to 2024. Our primary focus was on two main areas: Rehabilitation Robotics and Technology and Digital Twins (DTs). We also conducted a targeted examination of DTs in healthcare to assess their evolving role in medical research and clinical practice.

### 4.1. Rehabilitation Robotics and Technology

**Figure 4:**
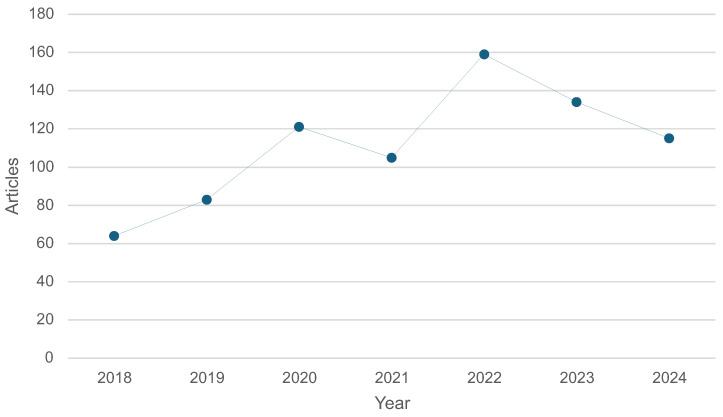
Annual scientific production related to rehabilitation robotics and technology from 2018 to 2024.

Using the Bibliometrix R package in RStudio, we queried PubMed for articles on “Rehabilitation Robotics” and “Technology”. As shown in Figure 4, the number of publications steadily increased, peaking in 2022. In particular, research focused on *human–robot interactions* for middle-aged adults, often in post-surgical recovery, stroke rehabilitation, gait disorders, paralysis, and balance training.

Many of these studies aimed to improve patient outcomes by combining robotic devices with sensors and AI-based algorithms. This integration enables real-time monitoring and feedback to guide patient motor relearning as well as remote or telerehabilitation approaches that reduce travel and healthcare burdens.

### 4.2. Digital Twins in Multiple Sectors

**Figure 5:**
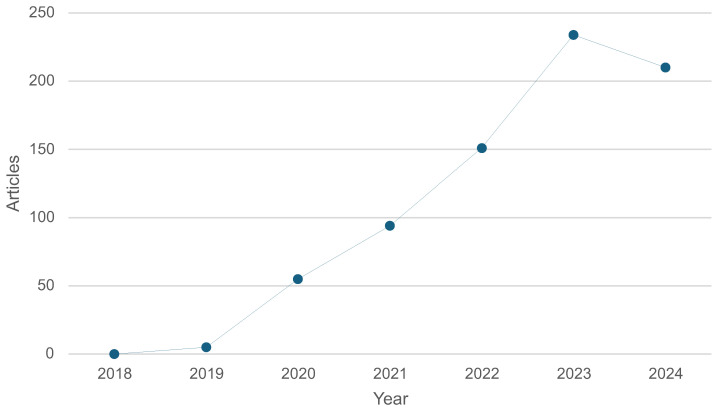
Annual scientific production related to digital twins from 2018 to 2024. The chart shows a rapid increase in the number of articles starting around 2020, reflecting a growing interest and research focus on digital twin technology.

We then extended our search to encompass overall DT research. From 2018 to 2024, the results showed a total of 748 articles, yielding a 30.66% annual growth rate. Figure 5 demonstrates a marked increase in DT-related publications beginning in 2020, with a peak of nearly 233 articles in 2023. This trend reflects growing enthusiasm for DT implementations that integrate artificial intelligence, machine learning, and deep learning.

In addition to healthcare, DT applications extend to agriculture, communications, and industrial manufacturing, among other domains. Although there is a growing body of work around “rehabilitation robotics” and “digital twins”, relatively few studies have explicitly explored their combined use under an AI framework, underscoring a noteworthy gap in the current literature.

### 4.3. Digital Twins in Healthcare

**Figure 6:**
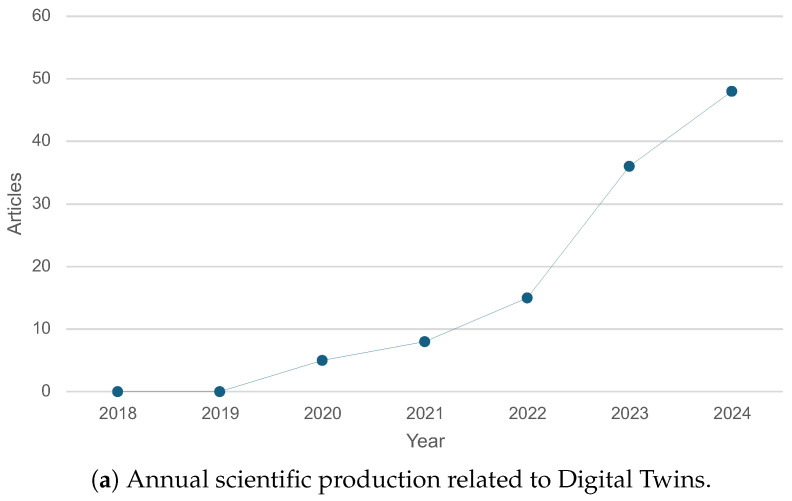

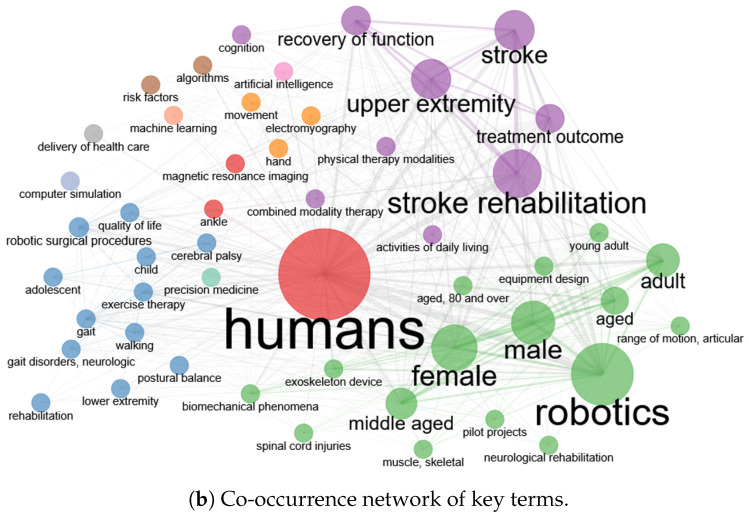
Digital Twins and Healthcare: (**a**) Annual scientific production related to digital twins in healthcare from 2020 to 2024, showing a peak of over 30 articles in 2023. (**b**) Co-occurrence network of key terms, emphasizing themes such as humans, AI, and healthcare delivery. Blue nodes indicate strong links to biomedical technology, nodes with warm colors cover AI and healthcare, and green nodes highlight population studied.

To gain a more targeted perspective, we specifically queried DTs in healthcare. As illustrated in Figure 6a, publications jumped to over 30 articles in 2023 alone, contributing to a total of 73 documents between 2020 and 2024 and an annual growth rate of 25.85%. The co-occurrence network in Figure 6b highlights the strong links between DTs, artificial intelligence, and healthcare delivery, with prominent topics including multiple sclerosis, magnetic resonance imaging, and pandemic-related challenges.

Current DT and AI research in the healthcare sector is characterized by a strong human-centric orientation, addressing specific challenges in rehabilitation, diagnosis, and response to health emergencies. As the co-occurrence network in Figure 6b shows, the high frequency of terms such as *humans*, *men*, *women*, *adults*, and *age* underlines the importance of tailoring interventions to population demographics. The prevalence of *stroke* as a primary focus area, along with *function recovery* and *upper extremity*, highlights the priority of restoring functional independence for patients. In this area, AI-driven *robotics* is emerging as a transformative tool for *rehabilitation*, enabling intensive and personalized therapies. By modeling the progression of recovery, DTs facilitate the simulation of robotic rehabilitation protocols and the optimization of individual outcomes.

In addition to rehabilitation, the utility of DTs and AI extends to the diagnosis and management of chronic diseases. The prominence of *magnetic resonance imaging* in Figure 6b is indicative of its crucial role as a high-fidelity data source for digital twins, allowing accurate modeling of pathologies such as *multiple sclerosis*. This technological integration enables the simulation of disease progression, the prediction of flares, and the optimization of individualized treatment plans in line with the principles of predictive and personalized medicine. Additionally, the relevance of *pandemic-related challenges* demonstrates the adaptability of these technologies to health crises. DTs can simulate the spread of diseases and the impact of public health interventions, while AI can process large-scale epidemiological data to predict outbreaks. This broad spectrum of applications ranging from post-stroke functional recovery to advanced diagnosis of chronic diseases and global health emergency preparedness cements the position of the patient at the heart of digital health innovation.

#### Core Journals and Bradford’s Law

**Figure 7:**
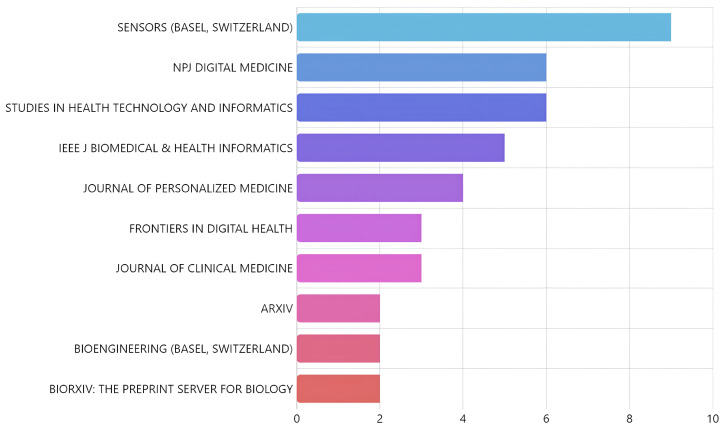
Core sources in digital twins and healthcare ranked according to Bradford’s Law. The decreasing frequency of articles across sources highlights that a small core of journals accounts for the majority of relevant publications.

We further used *Bradford’s Law* to identify core journals involved in DTs and the healthcare field. As seen in Figure 7, *Sensors* ranks highest, followed by *NPJ Digital Medicine*, *Studies in Health Technology and Informatics*, *IEEE Journal of Biomedical and Health Informatics*, and *Journal of Personalized Medicine*. This reflects a cross-disciplinary interest spanning sensor technology, health informatics, and personalized healthcare solutions.

As seen in Bradford’s Law, the distribution of publications identifies the essential journals that act as the main conduits for knowledge dissemination in this domain. The pre-eminence of Sensors is no coincidence; it reflects the intrinsic dependence of digital twins and AI on robust and continuous data collection. Without the ability to obtain high-quality physiological, environmental, or behavioral measurements through sensor technologies, the construction and validation of a digital twin would be unfeasible. Other core journals such as *NPJ Digital Medicine* and *Journal of Personalized Medicine* highlight the bias of research towards direct application in patient care and the tailoring of treatments to individual characteristics, which is a primary goal of DTs and AI in healthcare. Likewise, the presence of *Studies in Health Technology and Informatics* and *IEEE Journal of Biomedical and Health Informatics* emphasizes the technological and computational basis underpinning these advances, showing that much of the innovation comes from engineering and computer science applied to complex biomedical problems. This set of journals not only publishes research but also shapes the research agenda, fostering an ongoing dialogue between technology developers, informatics, and clinicians.

**Figure 8:**
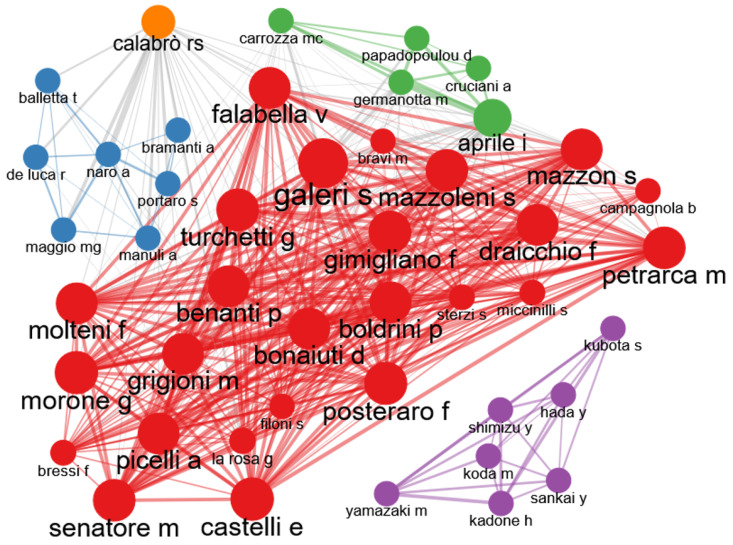
Collaboration network among the authors identified in the development of digital twins for rehabilitation.

Figure 8 illustrates the collaboration network among the authors, providing a detailed view of how knowledge is constructed and shared. The high density of interconnections within the core network attests to a highly collaborative research environment in which multiple experts combine their skills to tackle complex problems. The authors mentioned in the reference literature—such as Sosa-Méndez D. et al. with work in rehabilitation, or Wang W. et al. and Eminaga O. et al. exploring cancer detection and symptom triage systems—are examples of how inter-institutional and interdisciplinary collaboration drives innovation. Their contributions, which are likely to be scattered among the aforementioned core journals, demonstrate the practical application of DTs and AI in diverse clinical settings. However, the presence of a peripheral cluster (identified in purple) exhibiting significantly reduced connectivity represents a challenge. This topological isolation suggests that certain research groups or thematic areas may operate with a degree of independence; while allowing for specialization, this could limit cross-pollination of ideas and methodologies. Overcoming these barriers to collaboration through the promotion of research consortia, joint seminars and platforms, and the development of new research projects and methodologies can represent a key element in the development of new research projects and methodologies.

Research on digital twins and artificial intelligence in the healthcare field is strongly oriented towards practical and patient-centered solutions, with a particular emphasis on robotic rehabilitation and functional recovery after events such as stroke. While networked collaborations and publication in peer-reviewed journals are crucial to the advancement of the field, overcoming isolation barriers is also essential in maximizing the global impact of these technologies.

## 5. Applications of Digital Twins in the Health Field: A Critical Analysis

**Figure 9:**
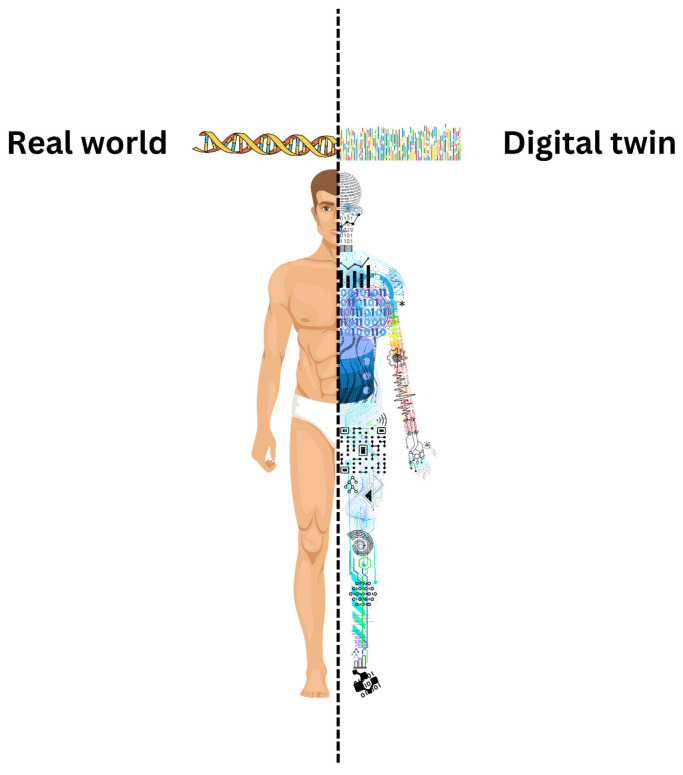
Digital twin, showing bidirectional composition between the real world and the virtual world.

Digital Twins (DTs) are becoming a cornerstone tool within healthcare systems. Their increasing adoption stems from the integration between large-scale data collection and predictive/diagnostic analytics, with both powered by Artificial Intelligence (AI) algorithms. These DTs provide a dynamic and evolving representation of a patient’s state, consistently synchronizing information with cloud-based platforms. Their scope is extensive and encompasses diverse physical, mechanical, and chemical variables of both individuals and the devices interacting with them. Human Digital Twins (HDTs) exemplify this approach by modeling not just physiological parameters but also psychological factors, creating a multifaceted view of human health [30]. These DTs are fed by sensors and biosensors that measure vital parameters, organ functionality, and mobility, among other indicators, then transmit them to a virtual model [31], as shown in Figure 9. In this way, clinicians can detect inherited genetic risks or interpret otherwise “normal” laboratory results in the context of behavioral data, enabling more accurate and timely diagnoses.

### 5.1. Critical Considerations: Addressing Data Heterogeneity and Privacy Protection in Healthcare Digital Twins

Despite the vast potential DTs offer to healthcare, their large-scale implementation faces critical challenges, particularly concerning data heterogeneity, privacy, and the need for real-time and low-latency processing. Addressing these limitations is imperative in ensuring the reliability and clinical utility of these innovative technologies. Data privacy constitutes a fundamental pillar; thus, sensitive medical information, biometric details, and other personal data must be rigorously protected in order to safeguard patient confidentiality and maintain trust. This is achieved through encryption, anonymization, and transparent communication with users regarding the collection, storage, and utilization of their data within the DT ecosystem [32,33]. For instance, wireless communication networks that transmit sensor data present inherent vulnerabilities, as noted by Zhiming Z. and colleagues [34], who proposed a homomorphic fingerprinting (HFPIDA) scheme to ensure integrity and authenticity in aggregated sensor data.

However, a more fundamental and intricate challenge lies in the heterogeneity of the data. DTs are fed by an amalgamation of sources, including electronic medical records, wearable devices, laboratory results, medical images, and even psychological and environmental data in the case of Human Digital Twins (HDTs). Each of these sources generates data in different formats and with varying frequencies and quality levels. This inherent diversity significantly complicates the integration and normalization of information, which can compromise the coherence of the digital twin and the ability to obtain a unified view of the patient’s health status. Without effective management of this data heterogeneity, the promise of DTs to offer a holistic representation of the patient cannot be fully realized.

To address these critical concerns, two promising technological approaches are gaining traction: Federated Learning (FL) and edge computing. *Federated learning* offers a robust solution for enhancing data privacy and security in DT updates. This method allows AI models to be trained collaboratively across multiple distributed data sources such as different hospitals or clinical sites without the sensitive raw patient data ever leaving the original location. Only aggregated model parameters or updates are shared and securely combined on a central server [35,36]. This approach is crucial for maintaining confidentiality and trust in healthcare, enabling continuous improvement and adaptation of digital twins with real-world data from diverse sources while adhering to strict privacy regulations.

*Complementing FL*, edge computing directly tackles the need for low-latency data processing and real-time responsiveness. By bringing computational power and data storage closer to the source of data generation (e.g., medical devices, sensors, or patient wearables), edge computing significantly minimizes the distance data must travel. This is vital for time-sensitive DT applications where immediate feedback is paramount, such as predictive monitoring, rehabilitation assistance, and critical decision-making in surgical procedures [37,38]. Edge computing not only reduces network congestion and improves operational efficiency, it also supports an agile lifecycle on the part of digital twins by facilitating near-instantaneous updates and simulations.

The synergy between federated learning and edge computing creates a powerful paradigm for advancing healthcare DTs. Edge devices can process local data and perform initial inferences, then leverage FL to securely contribute to the training and updating of global digital twin models. This integration ensures both real-time efficiency and scalability while upholding data privacy and fostering collaborative learning across a distributed healthcare ecosystem. This combined approach is poised to enable the development of truly intelligent, secure, and highly responsive digital twins that are capable of continuously adapting to individual patient conditions and dynamic environmental factors in an ethical and effective manner.

#### 5.1.1. Critical Analysis of AI-Supported Digital Twin Architectures

The evolution of digital twins in healthcare fundamentally depends on advanced AI architectures that can handle the complexity and diversity of medical data while maintaining clinical accuracy and reliability. Hybrid AI architectures combining deep learning with symbolic reasoning are emerging as particularly promising for healthcare applications, as they can simultaneously handle pattern recognition tasks such as medical image analysis or biosignal processing with the logical reasoning required for clinical decision-making [39]. These architectures address a critical limitation of pure deep learning approaches, namely, that they lack the explainability and interpretability that is essential for clinical acceptance and regulatory compliance [40].

Multimodal AI integration represents a crucial advancement for healthcare digital twins, enabling the simultaneous processing and correlation of diverse data types including medical imaging, genomic data, electronic health records, and real-time sensor streams. Advanced transformer-based architectures with attention mechanisms can dynamically weigh the importance of different data modalities, creating more comprehensive patient representations [41,42]. However, the computational complexity of these architectures presents significant challenges for real-time applications, particularly in resource-constrained healthcare environments where energy efficiency and processing speed are paramount.

Neuromorphic computing architectures offer a revolutionary approach to healthcare digital twins by mimicking the human brain’s processing patterns, providing ultra-low power consumption for continuous monitoring applications. These architectures show particular promise for implantable medical devices and long-term wearable sensors that require extended operation without battery replacement [43,44]. The challenge lies in adapting these biologically-inspired architectures to handle the precision requirements of medical applications while maintaining their inherent energy efficiency advantages.

Graph Neural Networks (GNNs) are gaining recognition for their ability to model complex biological systems and patient interaction networks, particularly in understanding disease progression patterns and treatment response variations. GNNs can capture intricate relationships between physiological systems, genetic factors, environmental influences, and social determinants of health, providing a more holistic and interconnected view of patient health [45,46]. This approach is particularly valuable for understanding complex chronic diseases where multiple factors interact in nonlinear ways.

#### 5.1.2. Real-Time Communication Protocols for Healthcare Digital Twins

The clinical utility of digital twins in healthcare critically depends on robust real-time communication protocols that can meet the stringent requirements of medical applications where latency, reliability, and security are paramount. Ultra-Reliable Low-Latency Communication (URLLC) protocols, originally developed for 5G networks [47], are being specifically adapted for healthcare applications where millisecond-level delays can have life-threatening consequences [39,48]. These protocols must guarantee bounded latency while maintaining 99.99% reliability, particularly for applications such as remote robotic surgery or real-time patient monitoring in intensive care units.

Time-Sensitive Networking (TSN) protocols are becoming essential for synchronized data collection from multiple medical devices and sensors within healthcare digital twin ecosystems. TSN provides deterministic communication with mathematically guaranteed bounded latency, which is crucial for applications requiring precise timing coordination between multiple systems such as robotic-assisted surgery or synchronized multimodal monitoring [49,50]. The integration of TSN with existing hospital networks presents significant technical challenges, particularly regarding legacy system compatibility and the substantial infrastructure investments required for network upgrades.

Blockchain-based communication protocols are being explored for ensuring data integrity and creating immutable audit trails for healthcare digital twins, addressing regulatory requirements for data provenance and patient safety [51,52]. These protocols can provide cryptographic proof of data authenticity and enable secure multi-party computation for collaborative healthcare scenarios involving multiple institutions. However, the inherent energy consumption and throughput limitations of traditional blockchain protocols necessitate the development of more efficient consensus mechanisms specifically tailored for healthcare applications, where sustainability and scalability are increasingly important considerations.

Edge-to-cloud communication protocols are evolving to support hierarchical processing architectures in which immediate decisions are made at the edge and complex analyses are performed in the cloud. Enhanced versions of protocols such as Message Queuing Telemetry Transport (MQTT) and Constrained Application Protocol (CoAP) are being developed with medical-grade security features, quality-of-service guarantees, and fault tolerance mechanisms [53,54]. These protocols must balance the need for immediate response with the requirement for comprehensive data analysis, creating a complex optimization problem that varies depending on the specific clinical application.

#### 5.1.3. Implementation Challenges and Critical Limitations

Despite these technological advances, several critical challenges threaten the successful implementation of AI-supported healthcare digital twins. Interoperability between different AI architectures and communication protocols remains a fundamental barrier to creating seamless healthcare ecosystems. The lack of standardized interfaces, data formats, and communication protocols continues to create isolated systems that cannot effectively share information or collaborate [55,56]. This fragmentation undermines the potential benefits of digital twins and limits their scalability across different healthcare providers and systems.

Real-time validation and verification of AI-driven decisions in healthcare digital twins requires new methodologies that can provide mathematical guarantees about system behavior under various conditions and failure scenarios. This is particularly challenging when dealing with adaptive AI systems that continuously learn and evolve, as traditional verification methods may not apply to systems that change their behavior over time [57]. The development of formal verification methods for dynamic AI systems represents a critical research gap that must be addressed before widespread clinical deployment.

Regulatory compliance presents another significant hurdle, as existing medical device regulations struggle to keep pace with the rapid evolution of AI technologies and communication protocols. Current regulatory frameworks were designed for static medical devices with predictable behavior, not for adaptive AI systems that learn and evolve in real-time [58,59]. New regulatory approaches specifically designed for AI-powered healthcare systems are urgently needed to ensure patient safety while enabling innovation.

Scalability and resource management concerns arise when considering the deployment of sophisticated AI architectures and communication protocols across diverse healthcare settings with varying technological capabilities and financial resources. The computational requirements of advanced AI models may exceed the capabilities of smaller healthcare facilities, potentially creating disparities in access to digital twin technologies [60,61]. Developing scalable solutions that can adapt to different resource constraints while maintaining clinical effectiveness represents a significant technical and economic challenge [62].

### 5.2. Towards Personalized Medicine: Challenges in Continuous Adaptation and Performance Management with Digital Twins

The transformative potential of DTs in healthcare lies in their ability to enable highly personalized and proactive medicine. By integrating continuous data from various sources, DTs promise to provide detailed insights that facilitate treatment adaptation, performance optimization, and prevention of adverse events. In contexts such as sports, for example, where the goal is to maintain athlete performance, prevent injuries, and respond proactively, DTs combine variables (such as sleep hours, caloric intake, heart rate, etc.) with Machine Learning (ML) techniques to generate personalized and adaptive plans [63]. This capability extends to monitoring physiological markers such as blood pressure and temperature to develop personalized goals and risk assessments in diverse populations [64,65].

However, the effectiveness of these personalized systems critically depends on the reliability and real-time adaptability of digital twin models. Although fatigue management in athletes has shown the value of AI in optimizing training intensity and minimizing overtraining [66], replicating this granularity and precision across the vast and complex spectrum of human health conditions presents significant challenges. Early detection of signs of overexertion, inadequate recovery, or the onset of chronic diseases demands impeccable synchronization between real-world metrics and virtual models. While the promise of precise real-time adjustments to treatment, nutrition, and recovery strategies is immense, its full realization requires overcoming the inherent limitations in continuously collecting, processing, and interpreting data within dynamic and highly individualized contexts.

### 5.3. From Monitoring to Personalized Treatment: Overcoming Real-Time Update Limitations and Algorithmic Biases

The utility of a DT in a clinical setting directly depends on its ability to reflect the patient’s state in real time. However, achieving and maintaining continuous high-fidelity synchronization with the physical world presents significant technical and computational constraints. The infrastructure required to constantly process and update large volumes of data from multiple sensors is complex and demands substantial computational capacity. Any latency in data transmission or processing could result in the digital twin representing an outdated patient state, which in turn might lead to erroneous diagnoses or to the application of ineffective or even detrimental treatment strategies.

Despite these limitations, integrating robust security measures with continuous physiological monitoring allows DTs to adapt and refine treatment strategies. For instance, remote monitoring systems can alert healthcare providers if a patient’s cardiac or respiratory status deviates from established thresholds, potentially triggering interventions before adverse events occur. Furthermore, the ability to analyze both current and historical data within a DT framework facilitates longitudinal studies, enabling healthcare professionals to uncover trends that might be overlooked in episodic evaluations. These capabilities deepen the potential of HDTs, transcending the mere replication of physiological parameters to incorporate holistic wellbeing, including mental health, lifestyle factors, and environmental conditions.

In the realm of prevention and diagnosis, Artificial Intelligence (AI) is a critical tool that allows for the integration of diverse patient data from symptoms to laboratory results, helping to produce increasingly accurate diagnoses and prognoses. However, these algorithms require large high-quality datasets that capture the full spectrum of clinical scenarios and individual variations in patient health. When combined with digital twins, AI can evaluate patient data in real time against extensive databases, enabling clinicians to compare current findings with medical evidence and prescribe personalized treatments [67]. An inherent challenge here is algorithmic bias, as the accuracy of AI predictions is directly linked to the quality and representativeness of its training data. If the data are not sufficiently diverse or reflect historical biases, then the digital twin could perpetuate or even amplify these disparities.

A clear example of this synergy is in cancer detection and management, where early detection often relies on nuanced signals that can be incorporated into classification algorithms [68]. In genomics, advanced computational tools have identified genomic markers associated with malignancies, such as the LASSO method for colon cancer [69] or deep neural networks for predicting survival rates in breast cancer [70]. In addition to oncology, AI-driven DT systems have proven valuable in cardiovascular diseases, although it has been observed that focusing solely on limited variables can overlook key disease-related patterns [71,72]. This highlights the importance of capturing a wide range of physiological and environmental inputs to fully leverage AI’s potential.

### 5.4. Innovation in Intervention: Digital Twins in Drug Development and Assisted Rehabilitation

Digital twins are increasingly influencing drug discovery and testing, providing a data-rich environment that can significantly reduce both costs and timelines. Through virtual modeling of biological systems, researchers can test how a new compound might interact with the human body, allowing them to narrow down potential drug candidates and refine their mechanisms of action without extensive in vitro or in vivo experiments [73]. This virtual simulation also facilitates drug repurposing. In the realm of in silico pharmacology, digital twins simulate drug interactions at the molecular, cellular, and organ scales, providing a nuanced understanding of efficacy and safety prior to real-world trials [74]. Additionally, DTs serve as a crucial tool for elucidating a drug’s mechanism of action, identifying the most promising candidates, and refining their design for maximum efficacy with minimal toxicity [75]. Finally, digital twins offer advantages in simulating clinical trial processes, enabling anticipation of complications and refinement of protocols prior to human exposure [76].

In addition to the general scope of drug development, a more specialized and crucial application of digital twins lies in personalized disease management, particularly for complex and highly individualized conditions. For example, the concept of intelligent digital twins is being explored for personalized patient care, aiming to predict disease progression and optimize interventions based on each patient’s unique physiological responses and lifestyle factors [77]. For the advancement of personalized therapeutic strategies, DTs are offering a vision in which patient-specific models integrate multi-omics data, clinical history, and treatment responses to simulate disease progression and predict optimal care pathways [78]. These applications highlight the ambitious goal of moving beyond general protocols toward truly individualized medicine, although they also underscore the considerable challenges in data integration, model validation, and ethical considerations for such complex patient profiles.

In the realm of movement rehabilitation, the COVID-19 pandemic significantly sped up the adoption of virtual patient interactions. Digital twins have been integrated with both supervised and unsupervised learning to recognize, classify, and predict movements and clinical states [79]. However, applying this to telerehabilitation remains challenging, especially when dealing with highly specific populations and validating performance in remote settings. Telerehabilitation interventions often require therapists to guide patients through individualized exercises, frequently with the help of wearable devices and webcams for real-time monitoring [80]. AI algorithms then analyze these data, providing insights and recommendations to adapt protocols.

Research has even extended rehabilitation to the cognitive domain by using facial expressions to assess emotional states or pain levels during motor tasks. However, its accuracy still needs refinement for large-scale clinical use [81]. Other work focuses on biomechanical evaluation such as using Inertial Measurement Units (IMUs) and deep neural networks to classify therapeutic exercises with high precision [82]. Advancing into robotics, a digital twin-based approach has been proposed to control the gait trajectory of the lower limbs, demonstrating that a DT model can effectively reproduce and guide physical movements with minimal error [19]. These studies point to a trend toward personalized and automated movement rehabilitation. Nonetheless, these systems still require validation in diverse patient groups and clinical conditions in order to truly transform telemedicine and remote rehabilitation. A critical challenge remains the scalability of these individualized expert methods to larger patient populations. While many technological contributions exist in rehabilitation, few systems use truly adaptive AI that can accommodate the wide range of patient characteristics and clinical presentations that make up a single disorder [83].

### 5.5. Continuous Monitoring and Assistance: Integrating DTs, IoT, and AI for Proactive Care

Digital twins are transforming patient care by enabling continuous real-time monitoring through wearable devices and other sensors. These virtual models are constantly updated to reflect changes in vital signs, activity levels, and other health metrics, which is especially valuable for managing chronic diseases requiring regular oversight [82,84]. Simultaneously, Internet of Things (IoT) technologies are being integrated into hospital workflows to reduce costs and unnecessary admissions. Compact devices equipped with biosensors and accelerometers transmit data to mobile platforms or cloud-based systems, allowing medical personnel to monitor patient activity and detect unusual behaviors or emergencies [85]. This approach has proven effective for identifying infections, predicting illnesses, and issuing alerts to those who have been in contact with infected individuals thanks to the precise data captured by wearable sensors [86,87].

Monitoring key physiological variables also enables the detection of potential musculoskeletal problems. For example, Huanxia [88] described a knee-strength recovery exoskeleton that incorporates a digital twin framework, ensuring the alignment of virtual and physical inputs for reliable control. By synthesizing data from multiple sources, digital twins support personalized care strategies that extend to nutrition tracking, medication management, and activity monitoring [82,84]. In telemedicine, these comprehensive virtual representations allow clinicians to make informed decisions without in-person consultations, boosting the accuracy and impact of remote healthcare interventions [89,90]. The integration of DTs with IoT and AI further refines predictive analytics, enabling early detection of impending health issues and automated alerts for care teams [84,91]. Such capabilities are particularly advantageous for home care of older adults or individuals with disabilities, as continuous remote monitoring minimizes rehospitalizations and can quickly drive targeted interventions [92].

Despite these clear benefits, challenges persist; for example, ensuring data security and privacy, optimizing interoperability with legacy healthcare systems, and managing the complexity of simultaneously collecting and processing information from diverse sensors represent significant hurdles. Overcoming these obstacles is crucial for wider adoption, as robust privacy measures and seamless data exchange are essential to preserving patient trust and maximizing the potential of digital twins in modern healthcare [92].

### 5.6. Future Trends and the Horizon of Digital Twins in Healthcare

Digital twin technology in healthcare is rapidly evolving, with medical professionals and researchers developing strategies to extend its reach beyond conventional settings. These virtual representations make it possible to design and monitor therapies, assess patient progress, facilitate diagnostic processes, and deliver personalized interventions even when the patient and clinician are geographically separated.

Current rehabilitation protocols often rely heavily on clinical devices or therapist-led interventions, which place limited physical demands on the patient; this is particularly true in passive rehabilitation scenarios, where the patient exerts minimal effort and might not fully engage in recovery exercises. In order to boost patient participation, researchers have introduced interactive feedback systems that collect quantitative data such as positional information, temperature, and humidity from wearable or integrated sensors [93]. These insights allow therapists to tailor individualized rehabilitation strategies that can accelerate recovery by adjusting exercises in real-time to the patient’s needs.

An example of this trend is the development of a specialized system that detects brain activity in stroke survivors to refine rehabilitation exercises [83]. However, the scalability of these individualized methods remains a challenge, as few systems employ truly adaptive AI capable of accommodating the wide range of patient characteristics and clinical presentations. In addition to rehabilitation, DTs show potential in remote and robot-assisted surgeries. A digital twin prototype has been presented for controlling a robotic arm in remote surgeries, highlighting the importance of secure communication, trajectory optimization, and robust cybersecurity measures for safe and effective teleoperated procedures [94].

Looking ahead, digital twins could transition from specialized clinical tools to widely available “digital coaches”. As these systems build upon expanding medical databases and real-time health metrics, they can offer personalized recommendations for nutrition, exercise regimens, and other healthy lifestyle changes. In this vision, DTs will serve not only high-performance athletes and patients with specific diagnoses but also the general population seeking to optimize physical and cognitive performance [83,95]. By leveraging continually updated large datasets, future DT platforms could provide real-time insights to users, enabling informed decisions about their daily habits and wellbeing.

## 6. Conclusions

Digital Twins (DTs) represent a transformative step forward in healthcare, offering precise modeling of patient physiology and enabling clinicians to deliver increasingly personalized and proactive care. As seen in their diverse applications from early disease detection and drug development to movement rehabilitation and athlete performance monitoring, the fusion of DTs with advanced sensing, Artificial Intelligence (AI) algorithms, and secure data management systems holds significant promise to improve patient outcomes.

By continuously integrating real-world data from wearables, in-home sensors, and clinical equipment, DTs can replicate complex physiological states and virtually test different treatment strategies prior to their real-world implementation. This approach has the potential to reduce adverse effects, streamline the rehabilitation process, and shorten overall recovery times. Importantly, insights generated from DTs can inform preventive measures, helping clinicians to detect risks earlier and tailor interventions more effectively.

Despite these advantages, several challenges remain. Data integrity, privacy, and interoperability across healthcare systems continue to pose major hurdles. Ensuring that DTs accurately reflect diverse patient populations also requires robust high-quality datasets and advanced modeling techniques. Generative models can help to fill this gap by creating synthetic data and facilitating scenario-based testing, ultimately enhancing the reliability of clinical predictions.

In general, DT research continues to expand by approximately 30% annually, and is making significant inroads into clinical practice. To fully harness the potential of DTs, future research should focus on improving simulation accuracy, integrating novel sensor technologies, and strengthening cybersecurity measures. Collaboration among stakeholders such as medical practitioners, data scientists, and policy-makers will be essential to translating the theoretical benefits of DTs into safe, efficient, and patient-centered healthcare worldwide. 

## Figures and Tables

**Table 1:** Types of Digital Twins (DTs) and their evolution in health. We considered three types of DTs: Digital Twin Prototypes (DTPs), Digital Twin Instances (DTIs), and Digital Twin Aggregations (DTAs).

Ref	Device	Goal	Software or Algorithms	Outcomes	Type of DT
Sosa-Méndez D. et al. [18]	Upper limb	Assess the upper limb rehabilitator’s function, simulating independent shoulder, elbow, and wrist movements.	Matlab/Simulink and SolidWorks	The robotic device is viable for advanced rehabilitation and can guide early-phase therapies using DTs, reducing costs by minimizing physical acquisitions.	DTP
Gao L. et al. [19]	Lower Limb Exoskeleton (LLE)	Synchronize motions between virtual and physical environments using the DDPG-PSO algorithm.	DDPG-PSO algorithms	A control strategy achieves a trajectory tracking error below 0.05 between the two environments.	DTA
Quinn A. et al. [20]	Robotic Knee Manipulator	Validate a robotic knee manipulator considering a DT and physical design.	OpenSim, SimBody, a C++ programming library, developed	configured the manipulator to reduce root-mean-square in flexion, adduction, and internal rotations movements with robotic DT.	DTI
Aluvalu R. et al. [21]	Emergency Room Symptomatology Identifier	Use an intelligent expert system to match patients’ symptoms with their digital records and relatives’ records in the cloud, aiming to reduce emergency department wait times and improve diagnosis accuracy.	–	Preliminary results show a success rate of over 80%, aiding medical staff and patients with expected diagnostic capabilities from data analysis.	DTP
Eminaga O. et al. [22]	Prostate cancer	Testing a DT using AI to detect prostate cancer through a database of 2603 images comparing six pathologies with the xPatho system.	xPatho system	xPatho identifies human pathology features but fails to develop a reliable DT for pathologists.	DTA
Wang W. et al. [23]	Gait Exoskeleton Robot	Relate active exoskeleton data to real patient needs at the simulation level.	–	Reliable exoskeleton gait results were obtained for a specific patient trajectory.	DTP
Sosa Méndez et al. [24]	Upper Limb Rehabilitation	Optimize the design of an upper limb rehabilitation device using a computerized method to advance to the construction.	SolidWorks and Matlab	The virtual model reduced the mechanism’s initial mass by 49% and proposed significant changes before construction and validation, with a maximum mean error of 0.11 rad.	DTP and DTI
Rahaman Khan M et al. [25]	Upper Limb Dysfunction	Offers remote therapy via an IIoT platform with a GUI and AR.	ThingWorx IIOT platform. Vuforia. Robots: xArm 5 and DMRbot. Exoskeleton: SREx	Performs 2D and 3D upper limb exercises with AR for telerehabilitation.	DTI
Maksymenko K et al. [26]	Myoelectrical digital twin	Training deep learning algorithms by simulating large amounts of data	Python API, ANN, Matlab	Cloud-based myoelectric DT simulating patient anatomy, motor unit traits, and muscle features.	DTA
Cruz Martínez G et al. [27]	Exoskeleton for passive rehabilitation of upper limb	Outline the phases and stages of the trajectory tracking methodology for a 7 DoF exoskeleton and validate it using a DT.	ERMIS exoskeleton, CPU SIEMENS 1500, Matlab - Simulink	Methodology for upper limb rehabilitation achieving 94.97% to 98.56% accuracy in trajectory tracking.	DTI
Lauer-Schmaltz et al. [28]	Avatar-based Human Digital Twins	Tore sensor information from the patient in a Human Digital Twin (HDT).	Graphical interface with avatar platform	User interface with animated avatar for caregivers to view patient exercises and therapy consultation dates.	DTI
Tao K. et al. [29]	Robotic interaction mechanism	Use AI to interpret visual stimuli and create adaptive trajectories for motor rehabilitation.	Visual cognition algorithms, facial expressions, and gestures.	AI-based robotic DT system for personalized learning.	DTA

## Data Availability

No new data were created or analyzed in this study. Data sharing is not applicable to this article.
